# Examining the views of operating room nurses and physicians on the relationship between professional values and professional communication

**DOI:** 10.1186/s12912-021-00778-x

**Published:** 2022-01-14

**Authors:** Sedigheh Yeganeh, Camellia Torabizadeh, Tayebeh Bahmani, Zahra Molazem, Hamed Yeganeh Doust, Samira Daneshvar Dehnavi

**Affiliations:** 1 School of Nursing, Gerash University of Medical Sciences, Gerash, Iran; 2 Community Based Psychiatric Care Research Center, Department of Nursing, School of Nursing and Midwifery, Shiraz University of Medical Sciences, Shiraz, Iran; 3 Department of Operating Room, School of Allied Medical Sciences, Fasa University of Medical Sciences, Fasa, Iran; 4 School of Nursing and Midwifery, Shiraz University of Medical Sciences, Shiraz, Iran; 5 Shahid Faghihi Hospital, Shiraz University of Medical Science, Shiraz, Iran; 6 Shahid Rajaei Cardiovascular Medical and Research Center, Tehran, Iran

**Keywords:** Professional communication, Professional values, Operating room nurses, Operating room physicians

## Abstract

**Purpose:**

Professional communication and professional values are two basic concepts in operating rooms and should be studied more closely in view of the nature of work and the high circulation of patients in operating rooms.

**Methods:**

The present work is a descriptive-analytic study with a cross-sectional design. The sample was 603 operating room physicians and personnel selected from the public hospitals of Shiraz. The data collection instruments were the 41-item professional communication questionnaire and the 26-item professional values scale.

**Results:**

The results showed that the operating room nurses and physicians perceived the status of professional communication and professional values to be satisfactory. As for professional communication, the participants’ perception of the domains of mutual respect and trust (*p* ≤ 0.001), teamwork (*p* ≤ 0.001), ethical competence (*p* ≤ 0.017), and workplace conflicts (*p* ≤ 0.001) was significant. As for professional values, only the dimension of care (*p* ≤ 0.016) was perceived to be significant. Moreover, a significant positive relationship was found to exist between professional communication and professional values (*p* ≤ 0.001).

**Conclusion:**

Considering the significance of the concept of professional communication and its connection with professional values, it is recommended that operating room personnel and physicians receive systematic education about professional communication and the harms of destructive attitudes as part of their academic education and afterwards.

## Introduction

Proper professional communication is characterized by “showing respect for professional values and personal abilities, relying on one’s colleagues’ knowledge and experience, and seeking advice before making decisions.” Establishment of proper professional communication in hospitals has always been a challenge in providing quality care to patients [1]. To reach their common goal, i.e., restoring their patients to good health within a certain period, the members of healthcare teams (physicians and nurses) need to observe the principles of professional communications, e.g. consulting each other and using each other’s experiences, especially in operating rooms where patients are at the greatest risk and time is limited [2, 3]. Studies show that poor communication caused by arrogance, verbal conflicts, cultural differences, interpersonal issues, unprofessional communication, stress and work overload, paperwork, dishonesty, etc. between members of healthcare teams in operating rooms can result in patient dissatisfaction and medical errors, e.g. retaining surgical items, operating on the wrong site, falling off beds, and increased fatality [4, 5] . According to Lari (2019), a large number of medical errors at the expense of patients are due to ineffective relationships between physicians and nurses [2]. Ineffective professional communication has consequences for healthcare teams too, including job dissatisfaction, repeated absence from work, inability to concentrate, loss of self-confidence, reluctance to participate in teamwork, and even violence against the other personnel [6, 7].

The cramped atmosphere of operating rooms, the personnel’s having close contact with each other for a long time in the same room, the high pressure of work, physicians’ tendency to make decisions without consulting the other members of the surgery team, nurses’ limited autonomy, and ethical inequalities in the medial personnel’s income and work privileges result in greater incidence of the above-mentioned consequences in operating rooms [2, 3, 8, 9].

In many hospitals, the relationship between physicians and the other personnel is not satisfactory, leading to implicit or explicit verbal conflicts and even physical fights which, inevitably, have adverse effects on the physicians, the personnel and, by extension, the patients. These clashes can undermine the personnel’s respect for ethical principles and professional values which are the foundations of nursing and medical practice and often determine the quality of care [3, 5, 10, 11].

In medical professions, professional values include benevolence, equality, freedom, human dignity, justice, and honesty. Connected with professional activities, professional values provide healthcare teams with a framework for decision-making [12, 13]. Professional values are adopted in interactive and acquisition-based processes which occur in professionals’ relationships with others in various situations. These processes are influenced by a variety of factors, including financial matters, past experiences, social groups and interactions in the workplace, personal characteristics, beliefs and values, and especially the dominant cultural context [12, 14–16]. Sometimes, the decisions made by physicians and nurses in an environment where the status of professional communication is poor lead to disagreement and value conflicts which cause one of the sides to suspect the core values of justice, benevolence, and equality and, consequently, to make less effort to promote professional values and, by extension, the quality of care. Professional values are closely connected with an individual’s personal beliefs and values and whatever is regarded as good for the members of the profession [13].

The medical personnel directly influence patients’ evaluation of clinical centers and medical services; thus, changes in professional values following strained relationships in medical environments can irreversibly damage patients’ trust in confidentiality and justice [11]. Due to overcrowding in the public hospitals of Shiraz, professional communication and professional values in the operating rooms are afflicted by many issues with serious consequences, including an increase in medical errors, reduction in self-confidence, poor concentration, perception of inequality, increased workload, unnecessary paperwork, increased immigration rates, and deterioration in the quality of care. In addition, hospitals do not have any systematic instruments or programs to evaluate and improve the personnel’s professional communication and professional values [17, 18] . Accordingly, the present study aims to identify the variables which correlate with professional communication and professional values as perceived by operating room doctors and personnel, to determine the status quo, to measure the compatibility of the criteria with the personnel’ responsibilities, and to suggest solutions to improve professional communication and perception of professional values.

### Research question

1. What is the relationship between professional communication and professional values in the operating room?
2. What is the status of professional communication and professional values in the operating room from the perspective of operating room nurses, Anastasia team and the surgical team?

## Method

The present study is a descriptive-analytic work with a cross-sectional design which was conducted in 2017. The study population consisted of 603 operating room nurses, anesthesia personnel and surgical (physicians) selected from 6 public hospitals in Shiraz using census sampling. The inclusion criteria were having an associate degree or bachelor’s degree in surgical nursing, nursing, or anesthesiology for the operating room personnel and at least a Doctor of Medicine degree for the physicians, willingness to participate in the study, and having at least 3 months’ experience of work in operating rooms. The data collection instruments were a demographics survey, the operating room personnel-physician professional communication questionnaire developed by Torabizadeh et al., and Weis and Schank’s professional values scale [4, 19].

The demographics survey consisted of 5 items. The professional communication questionnaire, developed by Torabizadeh et al., is comprised of 41 items which address 6 dimensions. Scoring is based on a 5-point Likert scale ranging from never to always with the score range being between 93 and 153. The validity and Cronbach alpha of the instrument are reported to be 0.92 and 0.88 respectively. A score of between 93 and 113 indicates poor relationship, between 113 and 133 indicates average relationship, and between 133 and 153 indicates good relationship [4]. Weis and Schank’s professional values scale is a standard survey with the score range of 26-130. The scale consists of 26 items which address five domains [19]. In Iran, the reliability of the scale has been calculated to be 0.92 [20]. In the present study, the sampling lasted for 3 months. The collected data were analyzed using IBM SPSS Statistics for Windows, Version 24.0, descriptive statistics, Pearson correlation, independent t-test, and one-way ANOVA.

### Findings

42.8% of the participants were male and 62% were married. The greatest frequency belonged to the age group 25-32 years. 53.1% of the participants were operating room nurses and 22.2% were physicians. Regarding work experience, the subjects who had 2 to 5 years’ experience (33.4%) constituted the largest group (Table 1).

**Table 1:** The study population’s demographic characteristics

Variable	Frequency	Percentage
Gender		
Female	345	57.2
Male	258	42.8
Marital status		
Single	229	38
Married	374	62
Age group		
< 24	59	9.8
25-32	293	48.5
33-40	156	25.9
41-48	59	9.9
> 49	34	5.6
Job category		
Operating room nurses	319	53.1
Anesthesia personnel	150	24.9
Surgical team	134	22/2
Work experience		
< 1	78	13
2-5	198	33.4
6-10	114	19.5
11-15	87	15.1
16-20	66	11.5
> 20	43	7.5

The results showed the professional communication mean scores of the operating room personnel and physicians to be 135.51 and 136.54 respectively, both of which indicate satisfactory communication. There was not a significant difference between the personnel’s and the physicians’ overall perceptions of professional communications. The participants’ professional communication mean scores were found to be significant in the domains of mutual respect and trust, teamwork, workplace conflicts (*p*≤0.001), and ethical competence (*p*≤0.017). From the nurses’ point of view, teamwork and workplace conflicts were relevant factors, while the physicians perceived mutual respect and trust and ethical competence to be relevant (Table 2).

**Table 2:** The means, standard deviations, and significance levels of the physicians’ and personnel’s professional communication total scores according to dimension

Dimensions of professional communications	Physicians Mean ± SD	Personnel Mean ± SD	***P***-value
Mutual respect and trust	54.68 ± 7.27	51.56 ± 8.93	0.001
Teamwork	19.79 ± 4.54	21.60 ± 5.27	0.001
Ethical competence	21.27 ± 3.43	20.41 ± 4.24	0.017
Workplace atmosphere	17.05 ± 2.15	16.73 ± 2.47	0.177
Workplace conflicts	13.44 ± 2.50	15.22 ± 2.61	0.001
Inter-professional interactions	10.29 ± 1.80	10.26 ± 1.84	0.892
Total score	136.54 ± 12.78	135.51 ± 14.55	0.46

Regarding professional values, there was not a significant difference between the nursing and anesthesia personnel’s and the physicians’ mean scores, except in the domain of care: the physicians perceived patient care as more important than the personnel did (*p*≤0.016). Overall, the participants’ mean scores showed that both the nursing and anesthesia personnel and the physicians considered professional values to be very important (Table 3).

**Table 3:** The means, standard deviations, and significance levels of the physicians’ and personnel’s professional values total scores according to dimension

Dimensions of professional values	Physicians Mean ± SD	Personnel Mean ± SD	***P***-value
Caring	35.49 ± 4.77	34.15 ± 7.35	0.016
Activism	19.47 ± 3.02	19.23 ± 3.64	0.459
Trust	20.04 ± 3.00	20.00 ± 3.27	0.897
Professionalism	15.36 ± 2.56	15.67 ± 2.88	0.24
Justice	12.08 ± 1.93	11.95 ± 2.15	0.514
Total score	102.47 ± 13.52	101.02 ± 16.92	0.321

To examine the relationship between professional communication and professional values, the researchers used Pearson’s correlation coefficient. The results showed that the correlation between the personnel’s professional communication mean score and professional values mean score was 0.36. As for the physicians, the correlation was 0.318. These figures are statistically significant considering the sample size of 603 individuals (*p*≤0.001), indicating that the better the status of professional communication between the members of healthcare teams, the more importance they attach to professional values.

With regard to whether demographic variables correlate with professional communication and professional values, the results showed that the physicians’ marital status and work experience and the personnel’s education did not affect their perceptions. However, the professional values mean scores of the two genders were significantly different (*p*≤0.016): the women attached greater importance to professional values than the men did.

Among the operating room nurses and anesthesia personnel, the professional communication mean score (*p*≤0.001) and professional values mean score (*p*≤0.004) of those whose work experience was one year or less and those whose work experience was between 2 and 5 years were significant: the personnel who had one year or less work experience considered professional values to be more important than the others did, while those with 2 to 5 years’ work experience regarded professional values as less important.

The results showed a correlation between the physicians’ educational level and their perception of professional communication and professional values. The physicians with higher education considered professional communication (*p*≤0.04) and professional values (*p*≤0.03) to be more important than the physicians with a lower educational level did.

## Discussion

The present study was conducted to investigate the relationship between professional values and professional communication as perceived by the operating room personnel and physicians at the public hospitals of Shiraz. 603 individuals participated in the study. The findings of the study showed that the operating room personnel and physicians perceived professional communication to be important, indicating that they considered professional communication to have a significant impact on the quality of care provided in the operating room. This attitude makes professionals feel obligated to have verbal and non-verbal communication with other professionals to minimize the rate of errors caused by inadequate communication. This finding is consistent with the results of the studies of Hailu and Norful [21, 22].

On the other hand, the participants’ responses showed that stress and heavy workload account for ineffective relationships in operating rooms, which is in keeping with the study of Halim [23]. Other contributory factors are assignment of too many responsibilities to one individual, workplace ergonomics, such as noise, too much reliance on paperwork for communication, and preoccupation with the main purpose of professional communications, i.e., caring for patients, at the cost of the other aspects of professional communications, including consulting others, transferring experiences, professional training, and teamwork. According to Bellandi (2018), physicians and nurses spend a large amount of their time filling out forms in hospitals [24, 25].

One of the domains of the professional communication questionnaire is teamwork, which includes support, cooperation, honesty, and satisfaction. The results of the present study show that the operating room nurses and anesthesia personnel attached more importance to the role of teamwork in professional communication than the physicians did, which is consistent with the study of Kwon (2020). Kwon reports that nurses have a higher opinion of teamwork than physicians do and have learned that teamwork helps reduce the frequency of medical errors, including burns, operating on the wrong site, retaining surgical items, and falls [26]. On the contrary, the results of the study of Gabriele Prati (2014) show that physicians have a better understanding of the significance of teamwork and claim to be more involved in teamwork activities [27]. It appears that the main reasons for the personnel’s greater emphasis on teamwork are differences between individuals’ professional perceptions and physician-centeredness in Iran: from the physicians’ point of view, since operations are performed by surgeons, teamwork in the operating room means being aware of the surgeons’ needs and following the surgeons’ instructions, not consulting others or transferring experiences [1, 28]. In addition, since the healthcare system in Iran belongs to the public sector and the hospitals in the present study were educational organizations, the surgeons in these hospitals are frequently replaced by other surgeons, while the other personnel’s stay lasts for many years. Accordingly, the physicians are likely to disregard the significance of teamwork and the personnel’s knowledge, which leads to the personnel’s frustration, burnout, and failure to share their experiences. In effect, the experienced personnel’s inability to transfer their experiences to new physicians is the outcome of past failures in their professional communications. János Kollár (2016) refers to ineffective professional communication as a contributory factor in medical personnel’s burnout [29]. Thekla Holmes (2019) introduces good teamwork as a primary contributor to personnel’s work satisfaction [28].

Another significant domain of the professional communication questionnaire is communication conflicts in the workplace, i.e., superiority, unprofessional and offensive behaviors, verbal abuse, and interpersonal issues. The results of the present study show that the operating room personnel considered conflicts as disruptive to professional communication more than the physicians did. According to the study of Laschinger (2014), physicians’ mistreatment of nurses in the workplace adversely affects nurses’ performance and undermines the quality of care and patients’ trust in nurses [30]. Based on the personnel’s responses to the items on the questionnaire (direct expression of requests, unprofessional communications, etc.), it appears that operating room personnel believe that, in the operating room, physicians and nurses experience two different kinds of professional communication: explicit (what happens when they meet each other) and implicit (their real impression of their professional communications). Nurses are not inclined to express their requests and issues openly and talk about their conflicts only in their implicit relationships, which can be due to fear of losing their jobs, not receiving support from their direct managers, and deficiencies in inter-professional communications. This condition (lack of proper communication) can endanger the patients’ safety [31]. According to Narelle Borrott (2017), because of their higher self-confidence and the nature of their profession, physicians find it easier to openly discuss their workplace requests and conflicts [32]. Replacing the course of social skills with a course on professional communication in the medical and bachelor’s degree curricula may help reduce the current issues [33].

Regarding ethical competence (religious differences, job status, cultural differences, leader-follower relationship, and pride), the results of the study show that the physicians perceived this dimension as more important than the nurses and anesthesia personnel did. The traditional nature of medical education and the absence of any training in professional communication in the past have inclined physicians to view the operating room personnel as subordinates who should merely obey them. They do not have a proper understanding of nurses’ status and consider the leader-follower relationship in their interactions with nurses to be acceptable. In other words, from the physicians’ point of view, operating room personnel should obey physicians without question, which is in compliance with mutual respect, a domain regarded highly by physicians [34] . The respondents (physicians and personnel) in the present study stated that religious and cultural differences were accepted in the workplace and there were not any conflicts in that area. As religious beliefs play an essential part in caring for patients, communicating with them, and maintaining their psychological health, it appears that absence of conflicts between physicians and the other personnel in religious matters can improve their professional communication [35].

As for the dimension of respect and mutual trust (respect for others’ opinions, mutual trust, and courtesy), the results of the present study show that the physicians perceived this dimension to be more important than the personnel did, which finding is consistent with the study of Esmaeilpour-Bandboni (2017). This study, which investigates physicians’ perception of their professional communication with nurses in Iran, reports that, in clinical procedures, physicians often trust nurses’ reports and rely on their insight in diagnoses and treatments. Physicians consider specialized knowledge, professional skill, and the ability to manage critical conditions as facilitators in professional communication and prefer to work with informed nurses [36–38]. It was also found that, regardless of their personality traits, physicians with higher education have a higher opinion of the value of professional communication and values, while, regardless of their personality traits, personnel with higher education are more likely to find themselves in conflicts.

In operating rooms, professional values are as important as professional communication. In the present study, the operating room personnel and physicians were found to perceive professional values to have an essential part in their professions, which finding is consistent with the studies of Poorchangizi (2019) [39], Torabizadeh et al. [40], and Domenico Montemurro (2013) [41]. Professional values are accepted and established criteria in medical sciences and physicians and nurses hold approximately similar views on them. According to Torabizadeh (2018), professional values are perceived similarly across different disciplines. However, physicians attach more significance to the caring dimension of professional values than nurses do, which is owing to the nature of their field and their responsibility for operations [24]. Also, compared to men, women perceived professional values to be more important, which is consistent with the study of Torabizadeh [40].

As core principles which determine the quality of care in operating rooms, professional values are closely related to professional communication: operating room nurses and physicians believe that better professional communication correlates with higher observance of professional values in operating rooms. Among the dimensions of professional values, caring for patients (reduction in errors and improvement in the quality of care), trust between the personnel and patients, and justice and professionalism (sharing professional experiences) improve when the status of professional communication is satisfactory. Moreover, proper professional communication in operating rooms can help the personnel improve their knowledge and skills and have a better understanding of professional values [42–44]. However, barriers to professional communication, including hierarchies, paperwork, disregard for teamwork, lack of a valid framework for dealing with communication issues, inequalities in the pay system in Iran, the dominance of a leader-follower relationship, unprofessional behaviors, and lack of education in professional communication in clinical environments, are undermining the significance of professional values for practitioners [29, 45].

Professional values have a complex nature, especially in the domains of justice and caring, and changes in these areas expose patients to hazards in ways over which they have no control. Even if it does not harm patients initially, poor professional communication can incline the personnel to attach less significance to professional values and change their attitude from being committed to benefitting patients to merely not causing harm to patients. The results of the present study show that operating room personnel’s commitment to professional values has diminished over time. According to Eriksson (2020), operating room nurses do not receive the respect which they deserve, which is often due to such issues as gaps between opinions and practice, occupational burnout, and lack of support in the workplace [46].

### Limitations of the study

Reluctance to participate was one of the main limitations of this study. The researchers tried to encourage participation by explaining that the results of this study could be a step towards solving professional communication problems in the near future.

## Conclusion

The results of the present study show that professional communication and professional values are related. However, most hospitals do not have any effective, systematic instruments or programs to evaluate and improve either area. It is recommended that hospitals develop systematic programs to train the personnel and assess their performance in professional communication and values free of any inter-disciplinary bias. Also, considering the essential role of these dimensions of professional practice, it is suggested that medical and paramedical students receive education in inter-professional communication in simulated conditions so that they will be able to establish better professional communication in real clinical environments.

## Data Availability

The datasets used and/or analyzed during the current study are available from the corresponding author upon reasonable request.
